# Technological Approaches for Neurorehabilitation: From Robotic Devices to Brain Stimulation and Beyond

**DOI:** 10.3389/fneur.2018.00212

**Published:** 2018-04-09

**Authors:** Marianna Semprini, Matteo Laffranchi, Vittorio Sanguineti, Laura Avanzino, Roberto De Icco, Lorenzo De Michieli, Michela Chiappalone

**Affiliations:** ^1^Rehab Technologies, Istituto Italiano di Tecnologia, Genova, Italy; ^2^Department of Informatics, Bioengineering, Robotics and Systems Engineering (DIBRIS), University of Genova, Genova, Italy; ^3^Section of Human Physiology, Department of Experimental Medicine (DIMES), University of Genova, Genova, Italy; ^4^Department of Neurology and Neurorehabilitation, Istituto Neurologico Nazionale C. Mondino, Pavia, Italy; ^5^Department of Brain and Behavioral Sciences, University of Pavia, Pavia, Italy

**Keywords:** brain–computer interface, motor impairment, neurologic disorder, neuromodulation, personalization

## Abstract

Neurological diseases causing motor/cognitive impairments are among the most common causes of adult-onset disability. More than one billion of people are affected worldwide, and this number is expected to increase in upcoming years, because of the rapidly aging population. The frequent lack of complete recovery makes it desirable to develop novel neurorehabilitative treatments, suited to the patients, and better targeting the specific disability. To date, rehabilitation therapy can be aided by the technological support of robotic-based therapy, non-invasive brain stimulation, and neural interfaces. In this perspective, we will review the above methods by referring to the most recent advances in each field. Then, we propose and discuss current and future approaches based on the combination of the above. As pointed out in the recent literature, by combining traditional rehabilitation techniques with neuromodulation, biofeedback recordings and/or novel robotic and wearable assistive devices, several studies have proven it is possible to sensibly improve the amount of recovery with respect to traditional treatments. We will then discuss the possible applied research directions to maximize the outcome of a neurorehabilitation therapy, which should include the personalization of the therapy based on patient and clinician needs and preferences.

## Introduction

According to the World Health Organization (WHO), neurological disorders and injuries account for the 6.3% of the global burden of disease (GBD) (1, 2). With more than 6% of DALY (disability-adjusted life years) in the world, neurological disorders represent one of the most widespread clinical condition. Among neurological disorders, more than half of the burden in DALYs is constituted by cerebral-vascular disease (55%), such as stroke. Stroke, together with spinal cord injury (SCI), accounts for 52% of the adult-onset disability and, over a billion people (i.e., about a 15% of the population worldwide) suffer from some form of disability (3). These numbers are likely to increase in the coming years due to the aging of the population (4), since disorders affecting people aged 60 years and older contribute to 23% of the total GBD (5).

Standard physical rehabilitation favors the functional recovery after stroke, as compared to no treatment (6). However, the functional recovery is not always satisfactory as only 20% of patients fully resume their social life and job activities (7). Hence, the need of more effective and patient-tailored rehabilitative approaches to maximize the functional outcome of neurological injuries as well as patients’ quality of life (8). Modern technological methodologies represent one of the most recent advances in neurorehabilitation, and an increasing body of evidence supports their role in the recovery from brain and/or medullary insults. This manuscript provides a perspective on how technologies and methodologies could be combined in order to maximize the outcome of neurorehabilitation.

## Current Systems and Therapeutic Approaches for Neurorehabilitation

The great progress made in interdisciplinary fields, such as neural engineering (9, 10), has allowed to investigate many neural mechanisms, by detecting and processing the neural signals at high spatio-temporal resolution, and by interfacing the nervous system with external devices, thus restoring neurological functions lost due to disease/injury. The progress continues in parallel to technological advancements. The last two decades there has seen a large proliferation of technological approaches for human rehabilitation, such as robots, wearable systems, brain stimulation, and virtual environments. In the next sections, we will focus on: robotic therapy, non-invasive brain stimulation (NIBS), and neural interfaces.

### Robotic Devices

Robots for neurorehabilitation are designed to support the administration of physical exercises to the upper or lower extremities, with the purpose of promoting neuro-motor recovery. This technology has a relatively long history, dating back to the early 1990s (11). Robot devices for rehabilitation differ widely in terms of mechanical design, number of degrees of freedom, and control architectures. As regards the mechanical design, robots may have either a single point of interaction (i.e., end effector) with the user body (endpoint robots or manipulanda) or multiple points of interaction (exoskeletons and wearable robots) (12).

**Figure 1:**
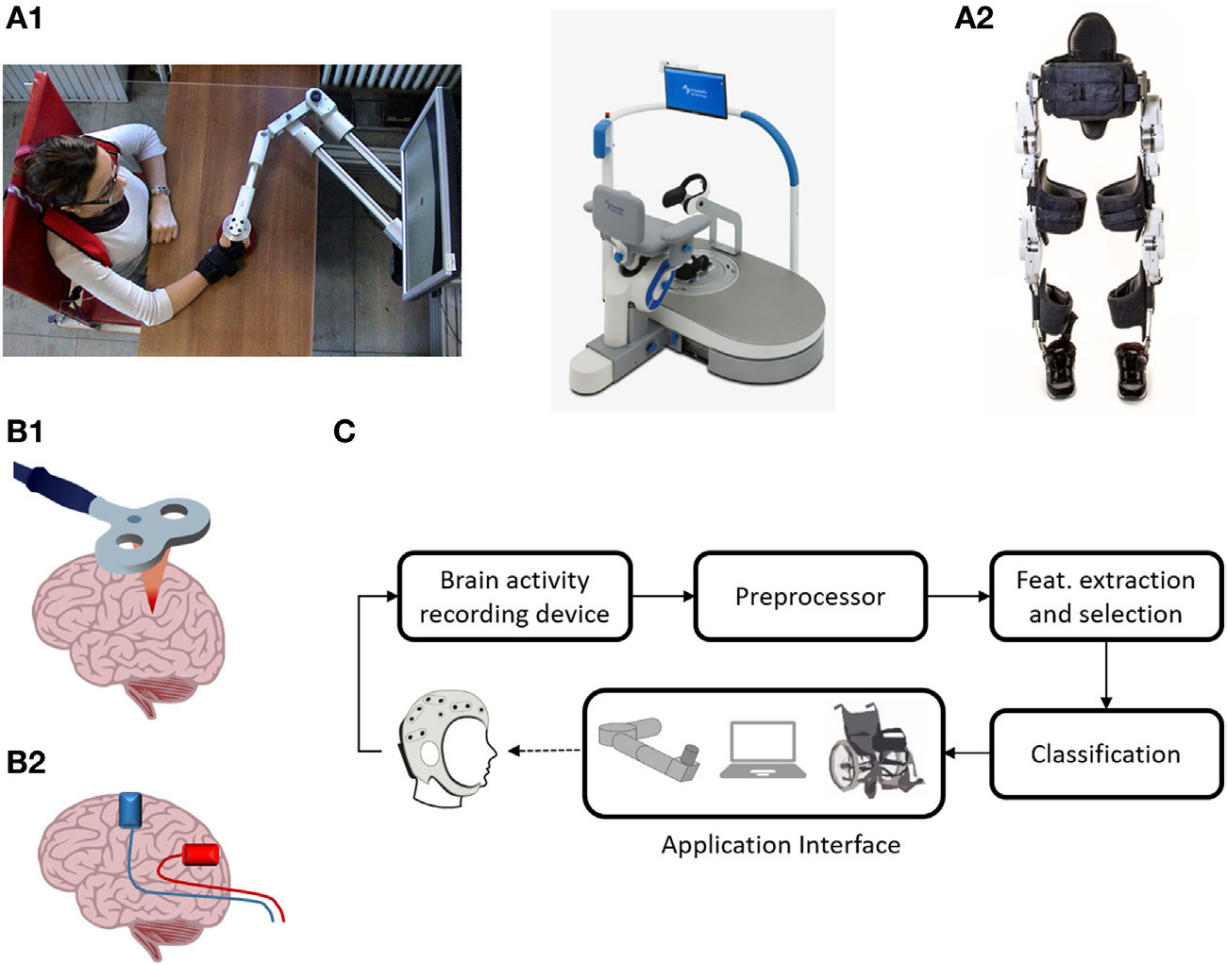
Neurorehabilitation therapies. **(A1)** Endpoint robots: on the left the “Braccio di Ferro” manipulandum, on the right the postural robot Hunova. Braccio di ferro (14) is a planar manipulandum with 2-DOF, developed at the University of Genoa (Italy). It is equipped with direct-drive brushless motors and is specially designed to minimize endpoint inertia. It uses the H3DAPI programming environment, which allows to share exercise protocol with other devices. Written informed consent was obtained from the subject depicted in the panel. Movendo Technology’s Hunova is a robotic device that permits full-body rehabilitation. It has two 2-DOF actuated and sensorized platforms located under the seat and on the floor level that allow it to rehabilitate several body districts, including lower limb (thanks to the floor-level platform), the core, and the back, using the platform located underneath the seat. Different patient categories (orthopedic, neurological, and geriatric) can be treated, and interact with the machine through a GUI based on serious games. **(A2)** Wearable device: the recent exoskeleton Twin. Twin is a fully modular device developed at IIT and co-funded by INAIL (the Italian National Institute for Insurance against Accidents at Work). The device can be easily assembled/disassembled by the patient/therapist. It provides total assistance to patients in the 5–95th percentile range with a weight up to 110 kg. Its modularity is implemented by eight quick release connectors, each located at both mechanical ends of each motor, that allow mechanical and electrical connection with the rest of the structure. It can implement three different walking patterns that can be fully customized according to the patient’s needs *via* a GUI on mobile device, thus enabling personalization of the therapy. Steps can be triggered *via* an IMU-based machine state controller. **(B1)** Repetitive transcranial magnetic stimulation (rTMS) representation. rTMS refers to the application of magnetic pulses in a repetitive mode. Conventional rTMS applied at low frequency (0.2–1 Hz) results in plastic inhibition of cortical excitability, whereas when it is applied at high frequency (≥5Hz), it leads to excitation (19). rTMS can also be applied in a “patterned mode.” Theta burst stimulation involves applying bursts of high frequency magnetic stimulation (three pulses at 50 Hz) repeated at intervals of 200 ms (20). Intermittent TBS increases cortical excitability for a period of 20–30 min, whereas continuous TBS leads to a suppression of cortical activity for approximately the same amount of time (20). **(B2)** Transcranial current stimulation (tCS) representation. tCS uses ultra-low intensity current, to manipulate the membrane potential of neurons and modulate spontaneous firing rates, but is insufficient on its own to discharge resting neurons or axons (21). tCS is an umbrella term for a number of brain modulating paradigms, such as transcranial direct current stimulation (22), transcranial alternating current stimulation (23), and transcranial random noise stimulation (24). **(C)** A typical BCI system. Five stages are represented: brain-signal acquisition, preprocessing, feature extraction/selection, classification, and application interface. In the first stage, brain-signal acquisition, suitable signals are acquired using an appropriate modality. Since the acquired signals are normally weak and contain noise (physiological and instrumental) and artifacts, preprocessing is needed, which is the second stage. In the third stage, some useful data or so-called “features” are extracted. These features, in the fourth stage, are classified using a suitable classifier. Finally, in the fifth stage, the classified signals are transmitted to a computer or other external devices for generating the desired control commands to the devices. In neurofeedback applications, the application interface is a real-time display of brain activity, which enables self-regulation of brain functions (25).

Endpoint robots for the upper extremity, include Inmotion2 (IMT, USA) (13), KINARM End-Point (BKIN, Canada), and Braccio di Ferro (14) (Figure 1A1, left). Only some of these devices have been tested in randomized clinical trials (15), confirming an improvement of upper limb motor function after stroke (16). However, convincing evidence in favor of significant changes in activities of daily living (ADL) indicators is lacking (17), possibly because performance in ADL is highly affected by hand functionality. A good example of lower limb endpoint robot is represented by gait trainer GT1 (Reha-Stim, Germany). Its efficacy was tested by Picelli et al. (18), who demonstrated an improvement in multiple clinical measures in subjects with Parkinson’s disease following robotic-assisted rehabilitation when compared to physical rehabilitation alone (18). Endpoint robots are also available for postural rehabilitation. For instance, Hunova (Movendo Technology, Italy, launched in 2017) is equipped with a seat and a platform that induce multidirectional movements to improve postural stability (Figure 1A1, right).

Typical lower limb exoskeletons range from large systems, equipped with treadmill and weight support, and intended for hospital use, like the Lokomat (Hocoma, Switzerland) and the LOPES system (26), to more lightweight devices intended for overground walking, like Ekso (Ekso Bionics, USA), Indego (Parker Hannafin, USA), Rewalk (Rewalk Robotics, USA), and the most recent one, Twin (IIT-INAIL, Italy). Notably, Twin has been developed according to long interactions with focus groups of disabled patients (Figure 1A2). A few exoskeletons for the upper limb have also been developed. They also range from lab systems—e.g., the KINARM Exoskeleton (BKIN, Canada) or the Armeo Spring and Power (Hocoma, Switzerland)—to wearable, modular devices (27–29).

One common feature of rehabilitation robots, is that they are equipped with movement and/or force sensors, so that they integrate functionalities both for the assessment [i.e., quantify users’ movements and exchanged forces (30)] and the treatment (i.e., administer highly reproducible, repetitive exercise protocols, and interaction modalities).

In spite of the increasing volume of published studies, the number of high-quality clinical trials on robot-assisted therapy is still relatively low. A large multi-center RCT comparing robot therapy, intensive physical therapy, and usual care (31) confirmed that robots are indeed effective, but found no significant advantage over conventional physical therapy. A systematic comparison of different approaches (32) suggested that robot therapy is among the most effective techniques for the rehabilitation of both upper and lower limbs. Moreover, recent studies concluded that robot-assisted gait training in combination with physiotherapy is more likely to achieve independent walking than gait training alone (33, 34).

A major limitation of endpoint robotic approach is that the improvement is limited to the body regions involved in training. In a clinical setting, robotic rehabilitation may be cost and time-consuming, and for this reason, it is difficult to imagine the combination of different endpoint robotic devices in the patient who have an impairment that affects multiple body areas, i.e., post-stroke hemiplegia. Moreover, early robots for neuroehabilitation were specifically aimed at substituting labor-intensive physical rehabilitation with minimal human intervention, producing an automatic and repetitive treatment. This initial trend, however, minimizes the importance of both therapist knowledge and patient–physician relationship. However, the ability to precisely quantify sensorimotor performance during exercise in terms of movement kinematics and exchanged forces is leading to a new revolution in rehabilitation, toward evidence-based and knowledge-driven approaches. Modern rehabilitation devices automatically adapt task difficulty and assistance modalities to individual performance (35). In the future, they may incorporate models of the recovery process (36) to predict the rehabilitation outcome (37) that will be fitted on patient’s features.

Another stimulating challenge is the development of lightweight robots suitable for the use outside of the hospitals, in domestic or community environments and in conjunction with ADL, e.g., over ground walking in unstructured environments. This implies a modular structure, which facilitates donning and transportability.

### Non-Invasive Brain Stimulation

Non-invasive brain stimulation techniques are a promising adjuvant strategy for enhancing post-injury recovery. In recent years, more than 1,400 studies were performed in humans, with at least one-fifth of these focusing on stroke rehabilitation. NIBS techniques involve modulation of the central nervous system by electrically activating neurons in the brain (38) and can be used to influence cortical excitability, neuroplasticity, and behavior (39, 40). Repetitive transcranial magnetic stimulation (rTMS, Figure 1B1) and transcranial current stimulation (tCS, Figure 1B2) are the most common and widely used techniques (39). Because of its relative ease of use, portability and decreased safety risk compared to rTMS, tCS is emerging as an effective and versatile clinical tool to prime the brain activity prior to or during neurorehabilitation. Starting from the hypothesis on training-induced plasticity, NIBS could be applied to foster plasticity induction, also in the spinal cord as shown in animals (41, 42) and in humans (43).

Related to rehabilitation, one of the major challenges is to design interventions that are efficient, promote motor learning, consolidate skills, and augment retention. For example, NIBS approach to stroke rehabilitation has focused on excitation of the unaffected hemisphere, of the affected hemisphere, or inhibition of unaffected hemisphere, also combining neuromodulation of both hemispheres (44). To date, a number of sham-controlled studies based on NIBS have been performed, but the evidence remains inconsistent. A Cochrane review failed to support the efficacy of rTMS for stroke rehabilitation (45), although other studies (46, 47) concluded that low frequency rTMS was effective in improving ADL and aphasia. A recent review (48) concluded that rTMS may produce both short- and long-term improvement on motor recovery in stroke patients, in particular when neuromodulation is initiated early after stroke, and with better results in case of sub-cortical lesions with respect to cortical ones. As regards tCS, it appeared to be useful for motor recovery in a sub population of patients with chronic stroke and low functional impairment (49) and very well tolerated (50), but a Cochrane review (51) failed to support its effectiveness. A possible explanation for these inconsistent conclusions is the lack of a correct patient stratification, and thus a tailored stimulation protocol (51, 52).

Some ethical and technical considerations deserve discussion. First, use of NIBS, calls for greater caution on pediatric population, given the higher stakes and uncertain future effects for brains still undergoing rapid and formative development (53). Second, a careful evaluation of the use of NIBS must also be warranted in adults, regarding informed consent and patient selection (54). Any direct interference with neural activity, even beneficial, might be described more accurately as “minimally invasive” (55). Moreover, when considering that most of the studies mainly focused on the short-term, short-lasting effects of NIBS, it is important to evaluate the long-term effects of modulating cortical electric fields in patients with cortical impairment.

Careful monitoring is particularly important when considering that, despite researchers’ discussion of and explicit warnings against unsupervised use of NIBS (56), brain stimulation products are already commercially available and without proper guidance or information. Thus NIBS could be conducted carelessly with unknown and potentially harmful effects.

### Neural Interfaces

In recent years, it is possible to include also neural interfaces among the strategies for neurorehabilitation and indeed the use of these systems in clinical applications is increasing (57, 58).

A neural interface is essentially a system mediating the communication between the brain and an external device (59, 60). Several modalities have been used for brain signal acquisition (61), which include electroencephalography (EEG) (62), magnetoencephalography (MEG) (63), functional magnetic resonance imaging (64), and functional near-infrared spectroscopy (65). Among neural interfaces, the so-called “BCIs” (Figure 1C) were essentially conceived as communication tools for paralyzed or locked-in patients (62) and were mainly based on the use of the processed EEG signal. Typical BCI techniques include the use of evoked potentials (such as P300) (66) or motor imagery (67), and enable the user to communicate with a speller device (68) or to control the movement of an end effector, either virtual (69) or real (70). From a clinical point of view, the BCI approach proves to be beneficial in potentiating the impaired motor function, as demonstrated for stroke (71, 72). MEG BCI training allowed patients with chronic stroke to voluntary modulate the μ-rhythm amplitude over the affected hemisphere with the possibility to voluntary control grasping using a robotic hand orthosis (73). More recently, a BCI-orthosis training was tested as add-on to physical therapy in a sham-controlled study (74); after an EEG BCI training protocol the strength in hand muscles significantly improved when compared to sham group. Noteworthy, the results of the above cited studies were achieved in patients in a chronic stage, for whom very limited possibilities are available if treated with standard rehabilitative care. The motor improvement is a consequence of the cortical changes occurring during the interaction with the controlled object, as demonstrated both with invasive and non-invasive studies (75, 76). This promising evidence made BCIs appealing for different types of neurorehabilitation practices, not only in presence of motor disability, but also for the recovery of impaired cognitive functions (76–80). In this framework, a particular form of BCI is that of neurofeedback, in which neural data are visually displayed to the user (81). This technique has proven to be mainly effective in the treatment of attention deficits/hyperactivity, but also for other cognitive dysfunctions (82, 83) and in stroke (84, 85).

Over the past decade also invasive brain-machine interfaces and neural prostheses in general have been the subject of extensive research with promising findings for the treatment of neuro-related impairments (86). The development of these devices will hopefully have a profound social impact on the quality of life, although translation to clinical application is far to be implemented due to the technological barriers (e.g., wired systems or limited bandwidth for wireless systems) and to the limits imposed by the invasiveness of the procedure (e.g., tissue reaction to the brain implant) (87).

Neural prosthesis can be combined with functional electrical stimulation (88, 89). In this scenario, the use of a controlled end effector is substituted by direct stimulation of the involved muscles, therefore, natural movements are recreated by bridging two areas disconnected because of the impairment/disease (88, 89). A system was recently developed allowing a quadriplegic patient chronically implanted with microwire arrays to move the arm by means of muscle stimulation triggered by the recorded and decoded brain signals (90).

Examples of latest-generation neural prostheses involve direct stimulation of central or peripheral neural tissue. Recent animal studies demonstrated locomotion restoration after SCI by spatiotemporal modulation of the spinal cord (91) and restoration of motor function after stroke by activity-dependent stimulation of the motor cortex (92). Whereas, recent human studies demonstrated the restoration of hand reaching and grasping by non-invasive neuromuscular stimulation of hand muscles (93) and prosthetic hand control by invasive stimulation of peripheral nerve (94). In addition, faster and more effective closed loop stimulation protocols are being investigated also in *in vitro* preparations (95).

## Challenges and Open Issues

**Figure 2:**
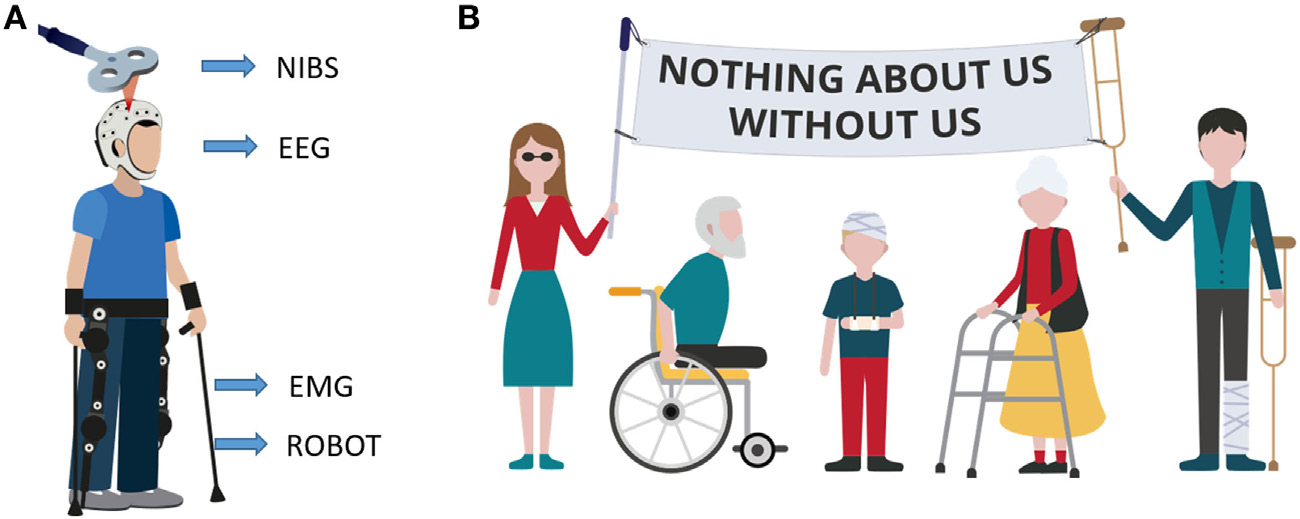
Innovative patient-tailored approach. **(A)** Example of multimodal rehabilitative approach. Subject is using an exoskeleton while receiving brain stimulation. Both exoskeleton motors and stimulation parameters are updated based on subject’s biofeedback signals (electroencephalography and/or EMG) and on subject performance (ROBOT) while, at the same time brain stimulation non-invasive brain stimulation and exoskeleton assistance (ROBOT) influence the biosignals. **(B)** Motto of the disabled population. The motto means that any choice (in any field) regarding them must be taken with their direct participation. Rehabilitation research must follow the same policy.

We have so far presented the main methodologies for neurorehabilitation and, for each field, the most innovative trends currently under investigation. However, novel rehabilitation approaches are characterized by a synergistic tactic, in which these techniques are used in combination and also mixed with kinematic information (from the robot) and patients’ biosignals, such as EEG or EMG (electromyography) (Figure 2A).

An example of such a multimodal approach was shown to the general public during the world cup in 2014, when the kick off was given by a paraplegic man using a lower limbs exoskeleton controlled by brain activity. Two years later, it was demonstrated that the combined use of gait rehabilitation with a BMI was able to induce partial neurological recovery in paraplegic patients (96). This represents a valid proof-of-concept for the combination of robotic devices driven by neural activity. Moreover, the number of clinical-oriented versions of this approach is increasing: exoskeletons powered by BCI have been used during post stroke rehabilitation (97, 98). Similar results were obtained with a BCI system for locomotion rehabilitation, based on the use of an avatar in a virtual reality environment (99).

Experiences where assisted locomotion has been used in conjunction with neuromodulation are already present in the literature. Spinal tDCS was applied in patients with SCI undergoing assisted locomotion using driven gait orthosis (Lokomat, Hocoma AG, Volketswil, Switzerland) (100). Results showed that anodal spinal tDCS and assisted locomotion increased spinal reflexes amplitude, suggesting functional effects when the spinal cord is detached from the rest of the central nervous system. These findings open an important avenue of research designed to rescue residual spinal functions by spinal tDCS in SCI patients (100).

Although the combined effect of neuromodulation with robotic therapy still needs to be clinically validated (101), it clearly shows the combined direction of neuromodulation-based rehabilitation. Explorative directions in neuromodulation also include a combination with traditional BMI approaches (102) as well as investigation of non-classical stimulation sites (103). Another potentially useful synergy in the rehabilitative field is represented by the association of biofeedback (104, 105) with robotic rehabilitation (106, 107).

Besides the technological and scientific improvement of neurorehabilitative treatment, a very important trend followed by current research is that of a *personalized treatment*. This is not just intended to focus on a particular disease and address the symptoms shared by different populations of patients, but is truly envisioned as a personalized method for a single individual. The idea of a patient-tailored approach is not new: standardized algorithms have been proposed for stroke, based on the clinical history of the patient, time elapsed after insult, topography of the lesion, type, and severity of functional impairment (108). In this view, it will be desirable to identify solid biomarkers not only in the acute settings, but also in the middle and chronic stages of neurological diseases. This modern approach, recently named as “Rehabilomics,” will be useful not only for outcome prediction, but also to foresee the best personalized rehabilitative treatment. Well known biomarkers in stroke are represented by measures of function and structure through neuroimaging after stroke (109) and by biochemical dosages (for example, uric acid, Cu/Zn superoxide dismutase, and urinary 8-OHdG) (110, 111). The technological improvement will help to identify novel biomarkers in neurorehabilitation. For example, a non-linear, composite, model made of robotic measurement in the upper limb was able to predict motor recovery at 90 days from stroke (112). “Technological” measures seem to be complementary rather than substitutive to standard biomarkers (113).

Overall, there is a great need for the development and testing of novel innovative interventional strategies individually tailored to patients’ prerequisites. The neurorehabilitation scientific community is finally showing an effort in this direction, by taking into account patients’ specific requests (Figure 2B). For example, during the sixth International Brain–Computer Interface Meeting in 2016 (114), a virtual forum of BCI users was presented, allowing patients to remotely participate at the conference sessions and also to send video-message with their views and requirements in order to help the scientists shaping the future research directions. This is exactly the approach that should be taken when designing a therapy or experimental protocol targeting a specific set of population. The patients’ motto “Nothing about us, without us” clearly indicates that patients must be involved in experimental studies, since the very beginning.

## Conclusion

In the coming years, science and medicine have to create a integrated dialog with patients, since they will be the first end-users of any technological development. To date, important advances have been made in robotic-based therapy, NIBS and neural interfaces, as integrations and/or alternatives to standard therapy. However, in order to be really effective, neurorehabilitation research must be primarily person-centered (i.e., “personalized”). Personalization calls for flexible solutions, such as combining the main technologies, in order to adapt to the different patient’s features and preferences. And this is exactly the direction which should be undertaken in neurorehabilitation.

## Author Contributions

MS, LDM, and MC conceived the manuscript. MC and LDM coordinated the activities. MS and MC prepared the figures. MS, MC, RDI, and LA revised the manuscript. All the authors wrote and approved the final version of the manuscript.

## Conflict of Interest Statement

The authors declare that the research was conducted in the absence of any commercial or financial relationships that could be construed as a potential conflict of interest. The handling Editor declared a shared affiliation, though no other collaboration, with one of the authors RI.
